# Association between dietary inflammation index and malnutrition status in peritoneal dialysis patients: a cross-sectional study

**DOI:** 10.3389/fnut.2025.1664787

**Published:** 2026-01-27

**Authors:** Jiaqian Zhong, Chuang Fan, Lin Li, Jiaming Wang, Huo Li, Zhongbo Bian, Hao Wang, Zhangming Pei, Hongchao Wang, Wenwei Lu, Juan Li

**Affiliations:** 1 Department of Nutrition, Shanghai Changzheng Hospital, Naval Medical University, Shanghai, China; 2 School of Public Health, Shanghai University of Traditional Chinese Medicine, Shanghai, China; 3 Bansongyuan Street Community Health Service Center, Shanghai, China; 4 Department of Nephrology, Shanghai Changzheng Hospital, Naval Medical University, Shanghai, China; 5 State Key Laboratory of Food Science and Resources, Jiangnan University, Wuxi, China; 6 School of Food Science and Technology, Jiangnan University, Wuxi, China; 7 National Engineering Research Center for Functional Food, Jiangnan University, Wuxi, China

**Keywords:** energy-adjusted dietary inflammation index, malnutrition, peritoneal dialysis patients, total fiber, cross-sectional study

## Abstract

**Objective:**

Chronic inflammation is prevalent in peritoneal dialysis (PD) patients, however, the potential impact of diet-related inflammation on PD patients has not been fully investigated. We aimed to explore the association between the Energy-adjusted dietary inflammatory index (E-DII) and malnutrition status in PD patients.

**Methods:**

A total of 147 PD patients from Shanghai Changzheng Hospital were included in this cross-sectional study. E-DII were calculated from the dietary data collected using a validated Food Frequency Questionnaire (FFQ). Malnutrition was determined according to the Malnutrition-Inflammation Score (MIS). Least absolute shrinkage and selection operator (LASSO) regression was carried out to screen the key nutrients associated with the risk of malnutrition. Univariate and multivariate logistic regression analyses were employed to explore the association between the key nutrients, E-DII and malnutrition.

**Results:**

The mean E-DII score was −0.367, ranging from *−*2.958 to 2.379. The mean duration of PD was 47.9 months. The overall prevalence of malnutrition was 48.3%. The key dietary nutrient associated with malnutrition was total fiber. In fully adjusted model, higher total fiber intake was associated with a lower risk of malnutrition in PD patients (OR_tertile_ 3 vs. 1 = 0.29, 95% CI: 0.10–0.80, *p* = 0.018). Conversely, a higher E-DII score was associated with a higher risk of malnutrition (OR_tertile_ 3 vs. 1 = 3.64, 95% CI: 1.25–10.64, *p* = 0.018).

**Conclusion:**

Diets high in total fiber were associated with a reduced risk of malnutrition in PD patients. On the other hand, pro-inflammatory diets are associated with an increased risk of malnutrition in PD patients. Further studies are needed to validate and develop strategies to reduce the dietary inflammatory burden in PD patients.

## 1 Introduction

The incidence of chronic kidney disease (CKD) is increasing due to the aging population and the increasing incidence of hypertension and diabetes mellitus globally every year (1–3). By 2020, there will be more than 697 million CKD patients globally (4). Peritoneal dialysis (PD) is one of the important treatments for end-stage kidney disease (ESKD). Although the prevalence of PD varies from country to country, PD patients account for approximately 11% of all dialysis patients (5). Malnutrition and inflammation are among the most common complications in PD patients worldwide (6), with a prevalence rate ranging from 30 to 50% in PD patients (7), and are among the most important risk factors affecting the health and prognosis of PD patients, not only severely affecting the quality of life of PD patients, but also significantly increasing the risk of all-cause and cardiovascular mortality (8–10).

Evaluating nutritional status is crucial for improving quality of life and clinical outcomes in patients undergoing maintenance peritoneal dialysis. The Malnutrition Inflammation Score (MIS) is an effective tool for assessing malnutrition and inflammation, and for the early screening of protein-energy wasting (PEW) in patients on PD. Evaluating nutritional status is crucial for improving quality of life and clinical outcomes in patients undergoing maintenance peritoneal dialysis. Studies have shown that the MIS is significantly and negatively correlated with nutritional and anthropometric indicators, and positively correlated with microinflammation indicators in PD patients (11, 12). MIS was also very effective in diagnosing PEW in PD patients, with an area under the ROC curve of 0.85. A cut off point of 7 (with 86% sensitivity and 75% specificity) was found to predict the onset of PEW in PD patients (13).

Diet may play an important role in the regulation of malnutrition and inflammation (14). Several recent systematic reviews and meta-analyses have indicated that dietary behaviors with high intakes of omega-3 fatty acids and zinc are associated with low incidence of malnutrition and inflammation (15–17). Peritoneal-dialysis patients are characteristically in a state of low-grade, persistent micro-inflammation that is closely linked to protein-energy wasting, renal anemia and adverse cardiovascular outcomes (18). Dietary Inflammatory Index (DII) has been established to quantify the overall inflammatory potential of the diet, based on the impact of different dietary components on inflammatory biomarkers (19). The DII is now widely used in clinical practice to investigate the potential relationship between dietary inflammatory potential and a variety of chronic diseases. In the context of CKD, diets with high DII scores may be a potential trigger for the development of malnutrition and inflammatory states in hemodialysis patients. An Australian prospective cohort study showed that higher DII scores were associated with poorer renal function at baseline and a greater decline in renal function over a 10-year period (20). Huang et al. (21) found that high DII was independently associated with all-cause and cardiovascular mortality over a 5-year period in patients with CKD. However, the association between DII and malnutrition in PD patients needs further exploration. The core strength of the energy-adjusted dietary inflammatory index (E-DII) over the traditional DII lies in its energy-standardization (22), which strips away the confounding effect of total caloric intake and thereby captures the intrinsic inflammatory potential of the diet rather than an illusion of “eating more or less.” Whereas the crude DII can be artificially diluted or inflated by high total energy intake, the E-DII is normalized per 1,000 kcal, ensuring comparability across individuals with widely differing energy intakes. In peritoneal-dialysis patients whose energy intakes vary widely, the E-DII is markedly better suited for both inter-individual and inter-study comparisons (23, 24).

As inflammation is one of the pathogenesis of malnutrition (25), and DII can reflect the inflammatory state in the body, it remains unclear if there is any relationship between DII and malnutrition in PD patients. Therefore, this cross-sectional study aimed to investigate the association between malnutrition and inflammatory potential of diets in PD patients by using the E-DII.

## 2 Materials and methods

### 2.1 Participants

The present cross-sectional study was carried out between April 2023 and October 2023 using PD patients from Shanghai Changzheng Hospital in China. Written informed consent was obtained from all participants before enrollment. The study protocol was approved by the Ethics Committee of Shanghai Changzheng Hospital, Ethics Approval No. 2023SL040. All PD patients were diagnosed with CKD by nephrologists and according to the criteria established by the expert group of the Shanghai Nephrology Clinical Quality Control Center, which included clinical characteristics, endoscopic, histological, and radiological examination et al. All PD patients started with intermittent peritoneal dialysis and gradually transitioned to continuous ambulatory peritoneal dialysis treatment after peritoneal dialysis placement.

The inclusion criteria were the following: (I) aged 18 to 80 years; (II) receiving maintenance peritoneal dialysis for more than 3 months; (III) regular follow-up and completion of peritoneal dialysis adequacy assessment. The exclusion criteria were the following: (I) those who were unwilling to participate and cooperate with the study; (II) those with serious data (demographic or laboratory biochemical) deficiencies; (III) those who were receiving weekly maintenance hemodialysis treatment in conjunction with peritoneal dialysis; (IV) those who had renal disease accompanied by malignant tumors, acute heart failure, cirrhosis, or immune system disease; (IV) those with renal disease accompanied with malignant tumors, acute heart failure, cirrhosis and immune system disorders; (V) history of surgery within the last 3 months; (VI) unconsciousness, communication disorders, and cognitive impairment. In addition, those on special diets (e.g., vegan, atkins) were also excluded from this study. Twenty patients were excluded due to missing values on the Food Frequency Questionnaire or other variables, resulting in a final sample of 147 patients. All researchers involved in this study received professional training.

### 2.2 Data collection

The following necessary information was collected through the interview and medical records: (I) sociodemographic data regarding age (years), gender (male/female), education (less than primary school/middle school/high school or equivalent/college or above), smoking status (current/former/never) and drinking status (yes/no); (II) body mass index was calculated as weight (kilograms) divided by height (m) squared and divided (BMI; kg/m^2^); (III) hypertension was defined as having been diagnosed by a physician or taking hypertension medication (yes/no), diabetes were defined as self-reported doctor diagnosis of diabetes, HbA1c ≥ 6.5%, FPG ≥7.0 mmol/L, postprandial 2-h plasma glucose ≥11.1 mmol/L from an oral glucose tolerance test or use of insulin or oral hypoglycemic medication (yes/no); (IV) serum albumin, serum creatinine, serum urea, uric acid, total cholesterol (TC), high-density cholesterol (HDL-C), potassium, phosphate, calcium and C-reactive protein (CRP). The estimated glomerular filtration rate (eGFR) scores were calculated using the Chronic Kidney Disease Epidemiology Collaboration algorithm (26).

### 2.3 Dietary intake measurement

Diet was assessed using the Food Frequency Questionnaire (FFQ). The questionnaire used in the present study included 27 groups of foods and beverages, which covered the majority of foods commonly consumed in China. Three aspects of each item were listed in the questionnaire, including whether the item was consumed, the usual frequency of consumption (number of times per day/week/month/year), and the estimated amount of food eaten each time, expressed using the local unit liang for weight (1 liang = 50 g) or cup for volume (1 cup = 250 mL). The translated version of the FFQ will be submitted as attachments Besides, nutrients and total energy intake were calculated by multiplying the usual frequency and portion size of each food item by the nutrient content using the 2018 6th Edition Chinese Food Composition (Standard Edition). In the calculation, Wincome’s hospital nutrition diagnosis and treatment system HNTS (V2.3.2.104) was used, and the 6th edition of “Chinese Food Ingredients” (Standard Edition) in 2018 was entered into the HNTS database. Then, the FFQ was added as a template to HNTS. When entering each person’s dietary data, entering whether they consume the item, the frequency of consumption, and the estimated amount of food consumed each time in the template can directly calculate the nutrients and total energy through HNTS.

### 2.4 Energy-adjusted dietary inflammatory index

The 26 foods and dietary components involved in DII were included in this study and are listed as follows: energy, protein, fat, carbohydrate, fiber, cholesterol, vitamins A, B1, B2, B3, B6, B12, C, D, E, iron, selenium, zinc, saturated fatty acids, monounsaturated fatty acids (MUFA), polyunsaturated fatty acids (PUFA), n-3 fatty acids, n-6 fatty acids, folate, and *β*-carotene. Dietary nutrient contents of each food were entered based on the data in the 2018 6th Edition Chinese Food Composition (Standard Edition).

According to the given DII scoring table and following the calculation method and logic of DII, use a self-designed calculation software to calculate the DII score of the research subjects. The specific calculation method for DII is as follows: for each participant in the study, first calculate the *Z*-score of DII for a certain dietary component. The *Z*-score is calculated by subtracting the global daily average intake from the average amount of dietary components and nutrients consumed by each participant during a day (midnight to midnight), and dividing this value by its standard deviation. Next, it was converted to a percentile score. To center the distribution with values, values were doubling and subtracting “1.” Finally, values were multiplied by “overall inflammatory effect score” and then summed to obtain the overall DII score (19). To adjust DII for energy, all dietary parameters were converted to reflect food intake per 1,000 calories (27). Ultimately, E-DII score was calculated using 25 food parameters and energy as a denominator.

### 2.5 Nutritional assessment

The Malnutrition Inflammation Score (MIS) was used to assess the nutritional status of the patients. The MIS consisted of 10 evaluation parameters in 4 parts, covering a range of physiological indexes and clinical manifestations of the patients, with the score of each parameter ranging from 0 (good condition) to 3 (severely poor condition), and the total score ranging from 0 (in normal condition) to 30 (in severely malnourished condition) (28). This study was designed with reference to existing research findings and accordingly set the cut-off value of poor nutritional status of patients. Specifically, when a patient’s MIS scale score was >7, the patient was judged to have poor nutritional status and was categorized into the poor nutritional status group, and when the score was ≤7, the patient was categorized into the good nutritional status group (28, 29).

### 2.6 Statistical analysis

SPSS Statistics 25.0 (IBM Corporation, New York, United States) and R version 4.3.0 (R Foundation for Statistical Computing, Vienna, Austria) were used for all statistical analyses. The entire statistical analysis process of this study, was conducted under the guidance and supervision of a professional statistician (HW, Department of Nutrition, Shanghai Changzheng Hospital, Naval Medical University, Shanghai 200003, China/attending doctor). HW was involved from the project initiation and experimental design stages, providing crucial support to ensure the rationality of the analysis plan. Continuous variables were expressed as mean ± standard deviation (SD), and categorical variables were described using frequency and percentage. ANOVA and chi-square test were used to identify the significant differences in variables data by the tertiles. Least absolute shrinkage and selection operator (LASSO) regression analysis was used to screen the variables by 10-fold cross validation to select the nutrients that contribute the most to EDII. Univariate and multivariate logistic regression was used to assess the association between the screened nutrients and malnutrition, in which the nutrient intake was classified into three groups from T1′ to T3′ and T1′ was used as the reference group. Univariate and multivariable logistic regression models were established to assess the association between E-DII and malnutrition. Participants were classified into tertiles based on E-DII, tertile 1 (T1) to tertile 3 (T3), T1 was used as the reference group.

## 3 Results

### 3.1 Baseline demographic characteristics

Of the 147 PD participants, 76 (51.7%) were female, and the mean age was 50.9 ± 12.7 years. The mean E-DII score was −0.367, ranging from *−*2.958 to 2.379. The mean MIS was 8.0 ± 3.7, among males, the mean MIS was 6.82 ± 2.70, while among females, the mean MIS was 9.03 ± 4.16; there were 71 patients with MIS >7 who suffered malnutrition, 26 males and 45 females. The mean duration of PD was 47.9 months. The average hemoglobin level was 106.3 g/L, and 121 (82.3%) patients were diagnosed with anemia. In terms of oedema legs, 36 patients (24.5%) had no edema, 39 patients (26.5%) had mild edema, and 72 patients (49%) had edema. The average circumference of the arm muscles was 22.7 mm, and the average skin fold thickness was 14.8 mm.

Table 1 shows the baseline characteristics of PD patients stratified by E-DII tertile. Compared with the patients in the first tertile (T1), patients in the third tertile (T3) who consumed a more pro-inflammatory diet have a higher prevalence of malnutrition and a lower blood potassium (*p* < 0.05).

**Table 1:** Baseline characteristics of PD patients by E-DII tertile.

Variables	Total (*n* = 147) (−2.958, 2.379)	T1 (*n* = 49) (−0.782, −0.075)	T2 (*n* = 49) (−0.161, 1.113)	T3 (*n* = 49) (0.129, 2.379)	*p*-value
Age (years)	50.9 ± 12.7	48.1 ± 10.1	52.2 ± 12.5	52.6 ± 14.9	0.153
Female (%)	76 (51.7)	28 (57.1)	28 (57.1)	20 (40.8)	0.175
Hypertension (%)	133 (90.5)	47 (95.9)	42 (85.7)	44 (89.8)	0.261
Diabetes (%)	25 (17.0)	8 (16.3)	7 (14.3)	10 (20.4)	0.714
Smoking status (%)					0.318
Current smoker	15 (10.2)	3 (6.1)	5 (10.2)	7 (14.3)	
Former smoker	16 (10.9)	3 (6.1)	8 (16.3)	5 (10.2)	
Nonsmoker	116 (78.9)	43 (87.8)	36 (73.5)	37 (75.5)	
Drinker (%)	5 (3.4)	1 (2.0)	3 (6.1)	1 (2.0)	0.619
Dialysis month (months)	47.9 ± 43.5	50.9 ± 40.3	53.0 ± 51.4	39.7 ± 37.4	0.271
BMI (kg/m^2^)	22.1 ± 3.6	22.2 ± 3.7	21.7 ± 3.2	22.6 ± 4.1	0.407
MIS	8.0 ± 3.7	7.1 ± 2.9	8.7 ± 3.2	8.1 ± 4.6	0.091
MIS-malnutrition (%)	71 (48.3)	16 (32.7)	31 (63.3)	24 (49.0)	0.010
Potassium (mmol/L)	4.1 ± 0.7	4.4 ± 0.8	4.0 ± 0.7	4.0 ± 0.6	0.012
Calcium (mmol/L)	2.3 ± 0.2	2.3 ± 0.2	2.3 ± 0.2	2.3 ± 0.2	0.827
Phosphorous (mmol/L)	1.8 ± 0.5	1.8 ± 0.5	1.7 ± 0.5	1.8 ± 0.5	0.329
Urea (mmol/L)	21.7 ± 6.6	22.9 ± 7.0	21.6 ± 5.9	20.7 ± 6.7	0.252
Uric acid (mmol/L)	390.6 ± 112.7	381.7 ± 113.8	402.4 ± 108.2	387.7 ± 117.1	0.649
Albumin (g/L)	33.3 ± 4.3	33.8 ± 4.2	33.5 ± 4.1	32.5 ± 4.7	0.301
eGFR (ml/min)	4.5 ± 2.3	4.2 ± 1.7	4.4 ± 1.9	4.8 ± 3.1	0.399
Creatinine (μmol/L)	1001.5 ± 321.5	1015.3 ± 296.9	969.6 ± 291.3	1019.5 ± 373.8	0.698
TC (mmol/L)	4.2 ± 1.1	4.3 ± 1.2	4.2 ± 1.1	4.0 ± 1.1	0.462
HDL-C (mmol/L)	1.0 ± 0.3	1.0 ± 0.3	1.0 ± 0.4	1.0 ± 0.4	0.811
C-reactive protein (mg/L)	8.6 ± 18.3	5.9 ± 9.5	6.7 ± 12.6	13.2 ± 27.1	0.095
Hemoglobin (g/L)	106.3 ± 19.2	110.9 ± 17.3	104.1 ± 19.1	103.8 ± 20.4	0.114
Anemia (%)	121 (82.3)	40 (81.6)	40 (81.6)	41 (83.7)	0.954
Oedema legs (%)					0.075
No edema	36 (24.5)	12 (24.5)	15 (30.6)	9 (18.4)	
Mild edema	39 (26.5)	15 (30.6)	6 (12.2)	18 (36.7)	
Edema	72 (49.0)	22 (44.9)	28 (57.1)	22 (44.9)	
AMC (mm)	22.7 ± 2.7	22.9 ± 2.4	22.4 ± 2.6	22.9 ± 3.0	0.636
Skin fold thickness (mm)	14.8 ± 4.7	15.3 ± 5.0	14.6 ± 5	14.6 ± 4.1	0.732

T1 = first tertile; T2 = second tertile; T3 = third tertile; E-DII, energy-adjusted dietary inflammatory index; BMI, body mass index; MIS, Malnutrition Inflammation Score; eGFR, estimated glomerular filtration rate; TC, total cholesterol; HDL-C, high-density cholesterol; AMC, arm muscle circumference.

### 3.2 Mean daily food categories and nutrients intake of PD patients in different E-DII tertiles

Table 2 shows the average daily intake of each food category in PD patients by E-DII tertile. We observed that PD patients in the first tertile had a higher consumption of vegetables (T3 vs. T1 = 212.4 ± 146.8 g/d vs. 452.7 ± 110.0 g/d, *p* < 0.001) and red meat (T3 vs. T1 = 52.8 ± 33.7 g/d vs. 76.2 ± 44.4 g/d, *p* = 0.012), compared with the patients in the third tertile.

**Table 2:** Dietary categories intake of PD patients by E-DII tertile.

Categories	Total (*n* = 147) (−2.958, 2.379)	T1 (*n* = 49) (−0.782, −0.075)	T2 (*n* = 49) (−0.161, 1.113)	T3 (*n* = 49) (0.129, 2.379)	*p*-value
Cereals-raw (g/d)	193.7 ± 70.3	190.7 ± 74.4	206.0 ± 67.6	184.4 ± 68.4	0.296
Potatoes (g/d)	27.5 ± 43.7	28.5 ± 48.8	23.2 ± 23.7	30.7 ± 53.2	0.689
Legumes (g/d)	29.5 ± 41. 8	28.0 ± 33.2	34.8 ± 54.3	25.7 ± 34.7	0.534
Vegetables (g/d)	316.5 ± 151.7	452.7 ± 110.0	284.4 ± 72.8	212.4 ± 146.8	<0.001
Fruits (g/d)	176.1 ± 136.1	208.1 ± 172.5	171.0 ± 114.8	149.2 ± 107.6	0.095
Dairy products (g/d)	140.0 ± 112.5	125.4 ± 106.7	133.3 ± 98.4	161.2 ± 129.1	0.255
White meat (g/d)	18.0 ± 19.0	17.4 ± 16.4	20.5 ± 24.5	16.0 ± 14.6	0.487
Red meat (g/d)	65.3 ± 46.1	76.2 ± 44.4	66.9 ± 55.4	52.8 ± 33.7	0.039
Fish and shrimps (g/d)	48.9 ± 45.8	56.2 ± 54.6	44.9 ± 34.4	45.4 ± 46.1	0.391
Eggs (g/d)	68.3 ± 48.1	80.4 ± 60.8	65.9 ± 42.4	58.8 ± 36.3	0.559
Liquids (g/d)	442.7 ± 282.9	421.0 ± 269.1	446.3 ± 265.5	460.7 ± 315.7	0.783

Table 3 shows the dietary nutrients intake of PD patients by E-DII tertile. We observed that PD patients in the first tertile had a high intake of anti-inflammatory nutrients, such as total fiber (T3 vs. T1: 6.4 ± 2.6 g/d vs. 11.2 ± 3.3 g/d, *p* < 0.001), n-3 fatty acids (T3 vs. T1: 0.3 ± 0.2 g/d vs. 0.4 ± 0.2 g/d, *p* = 0.023), folate (T3 vs. T1: 264.0 ± 103.0 μg/d vs. 146.2 ± 71.37 μg/d, *p* < 0.001), *β*-carotene (T3 vs. T1: 2487.0 ± 1146.1 μg/d vs. 7094.2 ± 1920.8 μg/d, *p* < 0.001), and vitamins such as vitamin A (T3 vs. T1: 423.7 ± 199.2 RE/d vs. 784.1 ± 210.4 RE/d, *p* < 0.001), vitamin C (T3 vs. T1: 109.4 ± 51.0 mg/d vs. 269.1 ± 76.1 mg/d, *p* < 0.001), vitamin B1 (T3 vs. T1: 0.5 ± 0.2 mg/d vs. 0.6 ± 0.3 mg/d, *p* = 0.001), vitamin B2 (T3 vs. T1: 0.7 ± 0.3 mg/d vs. 0.9 ± 0.3 mg/d, *p* = 0.003), vitamin B3 (T3 vs. T1: 13.3 ± 4.6 mg/d vs. 16.7 ± 5.4 mg/d, *p* = 0.004) and vitamin B6 (T3 vs. T1: 0.7 ± 0.3 mg/d vs. 0.9 ± 0.3 mg/d, *p* < 0.001), and minerals such as Mg (T3 vs. T1: 217.2 ± 61.3 mg/d vs. 306.0 ± 73.4 mg/d, *p* < 0.001), Zn (T3 vs. T1: 8.9 ± 2.8 mg/d vs. 10.9 ± 3.1 mg/d, *p* = 0.003) and Fe (T3 vs. T1: 15.8 ± 4.8 mg/d vs. 21.5 ± 6.0 mg/d, *p* < 0.001), compared with the patients in the third tertile.

**Table 3:** Nutrients intake of PD patients by E-DII tertile.

Variables	Total (*n* = 147) (−2.958, 2.379)	T1 (*n* = 49) (−0.782, −0.075)	T2 (*n* = 49) (−0.161, 1.113)	T3 (*n* = 49) (0.129, 2.379)	*p*-value
Energy (kcal/d)^a^	1491.3 ± 273.7	1471.9 ± 253.2	1498.0 ± 297.4	1504.1 ± 273.2	0.828
Protein (g/d)	63.6 ± 18.9	67.3 ± 18.0	63.8 ± 22.8	59.7 ± 14.5	0.140
Fat (g/d)	60.8 ± 12.3	58.9 ± 9.0	61.5 ± 12.6	62.1 ± 14.7	0.393
Carbohydrate (g/d)	166.3 ± 49.7	166.3 ± 52.8	169.9 ± 47.1	162.5 ± 49.6	0.766
Cholesterol (mg)	275.7 ± 222.7	290.5 ± 232.8	258.1 ± 223.7	278.5 ± 214.8	0.380
Total fiber (g/d)	8.6 ± 3.3	11.2 ± 3.3	8.2 ± 2.1	6.4 ± 2.6	<0.001
SFA (g/d)	12.1 ± 3.6	11.5 ± 2.7	12.0 ± 3.4	12.9 ± 4.4	0.134
PUFA (g/d)	22.9 ± 3.4	22.6 ± 2.6	23.5 ± 4.1	22.5 ± 3.3	0.254
MUFA (g/d)	14.1 ± 3.5	13.8 ± 2.6	14.2 ± 3.7	14.4 ± 4.0	0.686
n-3 Fatty acids (g/d)	0.3 ± 0.2	0.4 ± 0.2	0.3 ± 0.2	0.3 ± 0.2	0.023
n-6 Fatty acids (g/d)	1.5 ± 1.7	1.3 ± 0.9	1.6 ± 2.5	1.4 ± 1.2	0.811
Mg (mg/d)	261.4 ± 78.3	306.0 ± 73.4	261.1 ± 74.2	217.2 ± 61.3	<0.001
Zn (mg/d)	10.0 ± 3.3	10.9 ± 3.1	10.3 ± 3.6	8.9 ± 2.8	0.010
Fe (mg/d)	18.8 ± 6.0	21.5 ± 6.0	19.1 ± 5.9	15.8 ± 4.8	<0.001
Se (μg/d)	42.3 ± 19.3	45.8 ± 19.7	40.3 ± 17.9	40.8 ± 20.1	0.304
Vitamin E (mg/d)	39.9 ± 5.6	40.3 ± 4.7	40.9 ± 6.7	38.7 ± 5.2	0.129
Vitamin A (RE/d)	579.9 ± 244.4	784.1 ± 210.4	531.8 ± 167.2	423.7 ± 199.2	<0.001
Vitamin D (mg/d)	1.6 ± 1.5	1.8 ± 1.7	1.5 ± 1.2	1.6 ± 1.5	0.690
Vitamin C (mg/d)	184.0 ± 87.9	269.1 ± 76.1	173.4 ± 44.1	109.4 ± 51.0	<0.001
Vitamin B1 (mg/d)	0.5 ± 0.3	0.6 ± 0.3	0.5 ± 0.3	0.5 ± 0.2	0.004
Vitamin B2 (mg/d)	0.8 ± 0.3	0.9 ± 0.3	0.8 ± 0.3	0.7 ± 0.3	0.013
Vitamin B3 (mg/d)	15.4 ± 5.8	16.7 ± 5.4	16.3 ± 6.8	13.3 ± 4.6	0.008
Vitamin B6 (mg/d)	0.8 ± 0.3	0.9 ± 0.3	0.8 ± 0.4	0.7 ± 0.3	<0.001
Vitamin B12 (μg/d)	2.1 ± 1.8	2.4 ± 2.3	2.0 ± 1.4	2.0 ± 1.6	0.498
Folate (μg/d)	194.1 ± 91.5	264.0 ± 103.0	172.2 ± 44.5	146.2 ± 71.3	<0.001
β-Carotene (μg/d)	4635.9 ± 2384.1	7094.2 ± 1920.8	4326.3 ± 1142.5	2487.0 ± 1146.1	<0.001

T1 = first tertile; T2 = second tertile; T3 = third tertile; E-DII, energy-adjusted dietary inflammatory index; SFA, saturated fatty acids; PUFA, polyunsaturated fatty acids; MUFA, monounsaturated fatty acids.

All values have been adjusted for total energy intake using a residual method.

a Energy intake was not adjusted.

### 3.3 LASSO regression for key nutrients affecting malnutrition in PD patients

**Figure 1:**
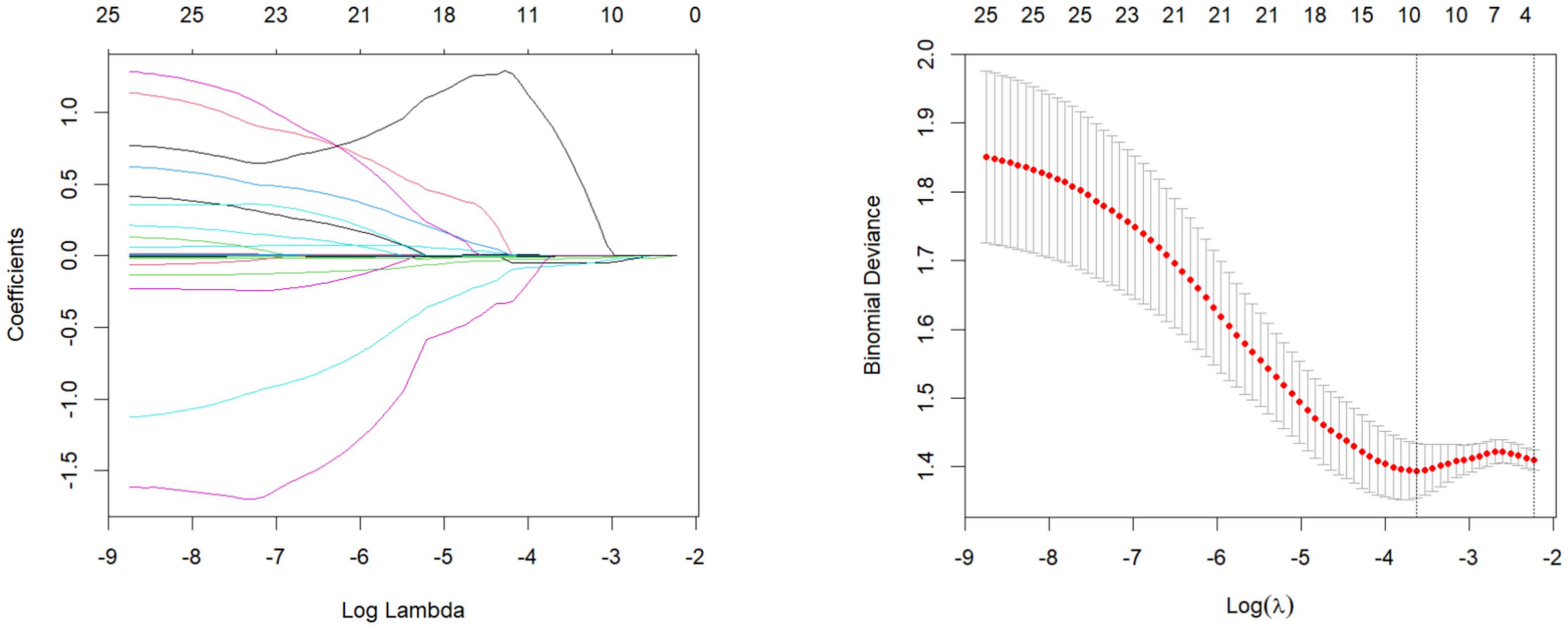
LASSO regression analysis to screen key dietary factors most related to malnutrition in PD patientslasso coefficient path diagrams and lasso regression analysis cross validation curves.

In the LASSO regression model, a total of 25 nutrients (adjusted by energy) were included, and the key nutrients were screened by 10-fold cross-validation. Figure 1A shows that the model error is the smallest when log(*λ*) = −3.628016, and the model screens out 10 variables. Figure 1B, each line represents a variable, the vertical axis is the coefficient of the parameter, and the horizontal axis is log(*λ*). The number of variables screened out varies with different values of log (*λ*), and the variables that have been screened out by LASSO have non-zero coefficients. In Figure 1B at *λ* = 0.026569, all 10 variables are retained in the model (with non-zero coefficients), and the coefficients of the rest of the variables are approaching zero with the increase of the penalty term. When *λ* is increased to 0.107259, the model is most appropriate for retaining only 1 variable. The LASSO regression model identified the key dietary nutrient associated with malnutrition was total fiber.

### 3.4 Logistic regression analysis for total fiber, E-DII and the risk of malnutrition in PD patients

**Figure 2:**
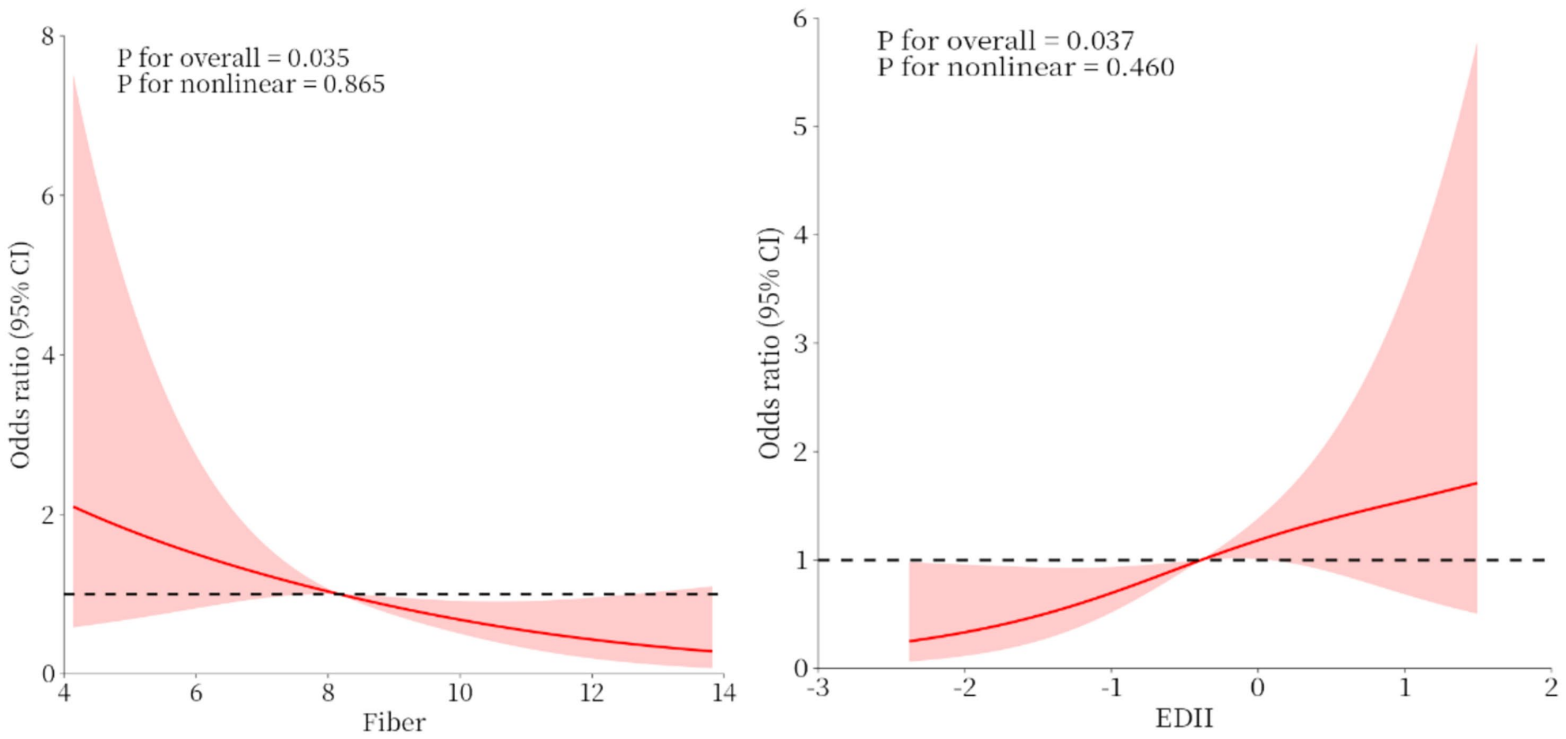
Restricted cubic spline fitting for the association between total fiber, E-DII and malnutrition.

The restricted cubic spline analysis revealed significant linear associations between both total fiber, E-DII intake and malnutrition risk (Figure 2).

The association between E-DII score, total fiber intake and malnutrition was further evaluated by univariate and multivariate logistic regression analysis (Table 4). We used total fiber as a continuous variable and divided the population into three groups using quartiles. The fiber intake of the first group T1′ was 2.4 g to 6.8 g, with an average of 5.4 ± 1.3 g. The fiber intake of the second group T2′ was 6.9 g to 9 g, with an average of 8.0 ± 0.6 g. The fiber intake of the third group T3′ was 9.1 g to 23.1 g, with an average of 12.3 ± 2.7 g. After adjustment for age, gender, smoking status, drinking status and BMI (Model 2), total fiber (as continues variable) was observed as a protective factor against malnutrition (OR = 0.84, 95% CI: 0.73–0.98, *p* = 0.025), and a positive association was observed between E-DII (as continues variable) and malnutrition (OR = 1.53, 95% CI: 1.07–2.19, *p* = 0.021). After further adjustment for hypertension, diabetes, dialysis month, eGFR, TC and HDL-C (Model 3), total fiber (as continues variables) remained a protective factor against malnutrition (OR = 0.81, 95% CI: 0.69–0.95, *p* = 0.010), specifically, the third tertile (T3) was associated with a lower risk of malnutrition in PD patients compared with the first tertile (T1) (OR_tertile_ 3 vs. 1 = 0.29, 95% CI: 0.10–0.80, *p* = 0.018); and a positive association was still observed between E-DII and malnutrition, with each unit increase in E-DII score was linked with 1.62 times increased odds of malnutrition (OR = 1.62, 95% CI: 1.11–2.37, *p* = 0.013), specifically, the third tertile (T3) was associated with a higher risk of malnutrition in PD patients compared with the first tertile (T1) (OR_tertile_ 3 vs. 1 = 3.64, 95% CI: 1.25–10.64, *p* = 0.018).

**Table 4:** Logistic regression analysis for E-DII, total fiber and malnutrition in PD patients.

Variable	Model 1		Model 2		Model 3	
	OR (95% CI)	*p*-value	OR (95% CI)	*p*-value	OR (95% CI)	*p*-value
Total fiber						
As continues	0.86 (0.76–0.97)	**0.012**	0.84 (0.73–0.98)	**0.025**	0. 81 (0.69–0.95)	**0.010**
T1′ (2.4 g, 6.8 g)	Ref		Ref		Ref	
T2′ (6.9 g, 9.0 g)	0.66 (0.30–1.47)	0.312	0.59 (0.23–1.49)	0.261	0.65 (0.24–1.78)	0.398
T3′ (9.1 g, 23.1 g)	0.40 (0.18–0.90)	**0.027**	0.31 (0.12–0.83)	**0.020**	0.29 (0.10–0.80)	**0.018**
E-DII						
As continues	1.24 (0.93–1.66)	0.140	1.53 (1.07–2.19)	**0.021**	1.62 (1.11–2.37)	**0.013**
T1 (−0.782, −0.075)	Ref	Ref	Ref			
T2 (−0.161, 1.113)	3.55 (1.54–8.17)	**0.003**	3.69 (1.41–9.66)	**0.008**	4.66 (1.65–13.19)	**0.004**
T3 (0.129, 2.379)	1.98 (0.87–4.49)	0.102	3.12 (1.16–8.41)	**0.024**	3.64 (1.25–10.64)	**0.018**

Model 1 was unadjusted. Model 2 was adjusted for age, gender, smoking status and drinking status, BMI. Model 3 was further adjusted for hypertension, diabetes, dialysis month, eGFR, TC and HDL-C. T1 = first tertile; T2 = second tertile; T3 = third tertile; BMI, body mass index; eGFR, estimated glomerular filtration rate; TC, total cholesterol; HDL-C, high-density cholesterol. Bold values indicate that *p* is less than 0.05 and has statistical significance.

## 4 Discussion

To the best of our knowledge, this is the first cross-sectional study to investigate the relationship between E-DII and malnutrition (MIS) in the Chinese population undergoing peritoneal dialysis. Malnutrition is common in PD patients, and it is associated with higher morbidity and mortality rates (30–32). In our study, the prevalence of malnutrition (MIS) was 48.3%, and we found that E-DII and total fiber intake is significantly associated with the reliable malnutrition marker MIS in PD patients.

Accurate assessment of the nutritional status of PD patients is a key component of clinical management (7). Routinely used tools include anthropometric indicators (e.g., body weight, BMI, triceps skinfold thickness, upper arm muscle circumference) and biochemical parameters (e.g., serum albumin, prealbumin, cholesterol) (33–35). However, there are significant limitations to the use of these indices in PD patients, as they are highly susceptible to strong interference from a variety of non-nutritional factors (36). Body weight and BMI are significantly affected by fluid loading status (e.g., volume of indwelling peritoneal dialysate, ultrafiltration insufficiency, water retention), which makes it difficult to reflect true lean body mass changes (37–39). Serum albumin, the most commonly used marker, is particularly limited in its value, not only as a nutritional indicator but also as a strongly negative acute phase response protein, whose synthesis is significantly inhibited by the chronic inflammatory state prevalent in PD patients (40); at the same time, persistent transperitoneal loss of albumin as well as humoral dilutional effects directly reduce its serum level (41). Similarly, prealbumin is inhibited by inflammation and its metabolism is influenced by renal function, and cholesterol levels correlate with inflammatory status and drugs (42–44). Therefore, relying solely on these traditional indicators to assess the nutritional status of PD patients is unreliable, and low levels tend to reflect inflammation, fluid disturbances, or protein loss more than purely inadequate nutritional intake. To overcome these limitations, the use of comprehensive assessment methods, such as the Subjective Global Assessment (SGA), MIS, specialized dietary intake analyses, bioelectrical impedance analyses (BIAs) performed under tightly controlled conditions, and grip strength measurements, allows for a more comprehensive identification and management of malnutrition risk in PD patients (45, 46).

Malnutrition is considered a complex state. PEW, which is prevalent in PD patients, is a highly complex pathophysiological state that occurs and develops as a result of multifactorial interactions, in which chronic inflammation plays a key role as an independent etiological factor and amplifies the negative effects of other factors, such as anorexia, acidosis, and insulin resistance, creating a vicious circle (7, 47). In addition, frequent infectious complications and reduced physical activity further exacerbate muscle wasting and deteriorating nutritional status (48, 49). The chronic inflammatory state of peritoneal dialysis patients is regulated by a variety of dietary factors, and unlike hemodialysis, peritoneal dialysis patients face more significant protein loss due to prolonged exposure to biologically incompatible dialysis fluids, and their protein intake needs to follow the principle of the U-shape relationship to avoid under- or over-consumption of protein (7, 50). Dietary fiber intake can produce short-chain fatty acids (SCFAs) (butyric acid, propionic acid) through fermentation by intestinal flora, inhibit histone deacetylase (HDAC), down-regulate the NF-κB pathway, and reduce the production of TNF-α and IL-6, as well as reduce uremic toxins such as indolephenol sulphate, and attenuate toxin-induced systemic inflammation (51). Polyphenols and flavonoids in plant foods have significant antioxidant and anti-inflammatory activities and may provide additional protection to PD patients (19, 52). Attention also needs to be paid to electrolyte balance such as sodium and phosphorus in peritoneal dialysis patients. In the present study, total dietary fiber, magnesium, iron, vitamin A, vitamin C, vitamin B1, vitamin B2, vitamin B3, vitamin B6, folic acid and *β*-carotene intake were significantly higher in the third quartile of E-DII. These results suggest a multifaceted relationship between diet, inflammation and malnutrition. The DII was formulated on the basis of the correlation between the potential inflammatory properties of the diet and six inflammatory biomarkers, including CRP, TNF-*α*, IL-10, IL-1β, IL-6 and IL-4 (52). In the current study, no correlation between E-DII and hs-CRP was observed. Consistent with previous studies, CRP is less sensitive to E-DII compared to other inflammatory biomarkers (19, 53, 54).

This study has several strengths. To the best of our knowledge, this is the first study that investigated the E-DII in relation to malnutrition in Chinese PD patients. Considering that the dietary habits and nutritional intake patterns of the Chinese population are different from those of Western countries, this study reflects the actual dietary patterns and nutritional status of Chinese PD patients through the use of dietary assessment tools and methods suitable for the Chinese population. Moreover, this study thoroughly analyzed the population-specific characteristics of Chinese PD patients, such as age, gender, prevalence of hypertension and diabetes mellitus, which may have an impact on the E-DII score and risk of malnutrition. For example, a higher proportion of Chinese PD patients had diabetes, which may have a more complex association with E-DII score and inflammatory status.

However, there were also some limitations in this study. Firstly, dietary intake was assessed by FFQ, which has limitations in terms of the potential for recall food groups. Secondly, this is a cross-sectional study with a small sample size, so the results should be interpreted with caution and we cannot infer causality. Thirdly, we calculated E-DII based on 25 dietary items, and data related to 19 items were not available in this study, which can affect the results. Finally, despite the adjustment for several known confounders, some residual confounding cannot be excluded in our findings; the E-DII captures only dietary-derived inflammation, overlooking dialysis-related triggers such as glucose degradation products (GDPs) in the dialysate and peritoneal infections—factors that can account for a sizeable proportion of the elevated inflammatory burden in PD patients (55). Multiple cross-sectional and retrospective cohort studies have demonstrated that micro-inflammation, assessed by classical biomarkers such as CRP, IL-6 and MIS, is significantly associated with malnutrition, hospitalization rates and cardiovascular events in PD patients; however, none of these investigations quantified dietary inflammation using E-DII (56–58).

## 5 Conclusion

This study showed that E-DII was significantly associated with MIS in PD patients, which not only fills the gap in the study of E-DII in PD patients in China, but also provides valuable data and insights into the field of PD. By revealing the relationship between E-DII and malnutrition in PD patients, it can help develop targeted nutritional interventions to improve the prognosis and quality of life of PD patients. However, further longitudinal studies are needed to infer a cause-and-effect relationship between E-DII and malnutrition in PD patients.

## Data Availability

The raw data supporting the conclusions of this article will be made available by the authors, without undue reservation.
